# A review on the bioleaching of toxic metal(loid)s from contaminated soil: Insight into the mechanism of action and the role of influencing factors

**DOI:** 10.3389/fmicb.2022.1049277

**Published:** 2022-12-07

**Authors:** Emmanuel Konadu Sarkodie, Luhua Jiang, Kewei Li, Jiejie Yang, Ziwen Guo, Jiaxin Shi, Yan Deng, Hongwei Liu, Huidan Jiang, Yili Liang, Huaqun Yin, Xueduan Liu

**Affiliations:** ^1^School of Minerals Processing and Bioengineering, Central South University, Changsha, China; ^2^Key Laboratory of Biometallurgy of Ministry of Education, Central South University, Changsha, China; ^3^Hunan Agricultural Biotechnology Research Institute, Hunan Academy of Agricultural Sciences, Changsha, China

**Keywords:** bioleaching, toxic metal(loid)s, contaminated soil, remediation mechanism, influence factors

## Abstract

The anthropogenic activities in agriculture, industrialization, mining, and metallurgy combined with the natural weathering of rocks, have led to severe contamination of soils by toxic metal(loid)s. In an attempt to remediate these polluted sites, a plethora of conventional approaches such as Solidification/Stabilization (S/S), soil washing, electrokinetic remediation, and chemical oxidation/reduction have been used for the immobilization and removal of toxic metal(loid)s in the soil. However, these conventional methods are associated with certain limitations. These limitations include high operational costs, high energy demands, post-waste disposal difficulties, and secondary pollution. Bioleaching has proven to be a promising alternative to these conventional approaches in removing toxic metal(loid)s from contaminated soil as it is cost-effective, environmentally friendly, and esthetically pleasing. The bioleaching process is influenced by factors including pH, temperature, oxygen, and carbon dioxide supply, as well as nutrients in the medium. It is crucial to monitor these parameters before and throughout the reaction since a change in any, for instance, pH during the reaction, can alter the microbial activity and, therefore, the rate of metal leaching. However, research on these influencing factors and recent innovations has brought significant progress in bioleaching over the years. This critical review, therefore, presents the current approaches to bioleaching and the mechanisms involved in removing toxic metal(loid)s from contaminated soil. We further examined and discussed the fundamental principles of various influencing factors that necessitate optimization in the bioleaching process. Additionally, the future perspectives on adding omics for bioleaching as an emerging technology are discussed.

## Introduction

Soil contamination is mainly caused by increased urbanization, rapid industrialization, agricultural practices, and inappropriate waste disposal methods. These activities generate solid wastes and wastewater that are toxic to the environment and pollute the soil, posing a threat to human and animal health (Ettler, 2016; Rodríguez-Eugenio et al., 2018; Zhang et al., 2020). Toxic metal(loid)s pollution of soil has become a global environmental issue yet to be resolved. In the United States, over 100,000 sites have been reported to have suffered from soil pollution, with Cd, Cu, Pb, Hg, Ni, and Zn being considered the most hazardous pollutant and are included in the US Environmental Protection Agency’s (USEPA) list of priority pollutants (He et al., 2015). The situation is not different in China; according to the Ministry of Environmental Protection and the Ministry of Land and Resources of China, the average contents of Cd, Hg, As, Cu, Pb, Cr, Zn, and Ni in the soil in China exceeded the regulatory levels by 7.0%, 1.6%, 2.7%, 2.1%, 1.5%, 1.1%, 0.9%, and 4.8%, respectively (Bulletin of National Soil Pollution Survey, 2014). Toxic metal(loid)s (Cd, As, Cr, Cu, Pb, Hg, Ni, and Zn) contamination in soils poses a major danger not only to soil quality and safety but also to animal and human health. The remediation of soils contaminated with toxic metal(loid)s is, therefore, crucial.

Currently, the most commonly used soil remediation technology is the application of a soil conditioner to passivate toxic metal(loids). However, the heavy metal passivation cannot reduce the soil’s toxic metal(loid)s content and decrease it to the standard rate, thus risking pollution rebound (Lan et al., 2021; Wang et al., 2021). Therefore, reducing the toxic metal(loid)s content in contaminated soil is very desirable and a pressing necessity. Despite the high extraction efficiencies of chemical, washing, and electrokinetic remediation, the practical implementation of these processes on a broader scale has its own set of constraints since they necessitate substantial investments in leaching reagents and operations (Mena et al., 2016; Zhai et al., 2018; Shen et al., 2019; Bhatt et al., 2021). Consequently, there is a growing demand for technologies to remove soil toxic metal(loid)s. The development of innovative low-cost, efficient, and environmental-friendly removal techniques has emerged as one of the key research fields. In recent years, bioleaching has gained much attention and has been proposed as a method for decontaminating solid wastes that include toxic metals in contaminated soil (Yang et al., 2018b). Bioleaching is a microbial-based method that relies on different microorganisms’ abilities to translate heavy metal fraction into soluble and extractable compounds that can be leached (Bosecker, 2001; Gu et al., 2018). Bioleaching toxic metal(loid)s from contaminated soil relies on two main strategies: immobilizing/stabilizing toxic metal(loid)s or mobilizing/extracting them with microorganisms.

Nevertheless, suppose the goal is to remove the toxic metal(loid)s and lower their concentration; in this case, the second strategy is preferred since the first approach just lessens the toxicity of the metal(loid)s by reducing their mobility while leaving some traces of the toxic metal(loid)s in the soil. Unlike chemical washing and electrokinetic remediation, bioleaching is an eco-friendly technique, does not require concentrated chemicals, emits no hazardous chemicals, and also economically feasible (Vyas and Ting, 2020). For instance, studies by Deng et al. (2013) revealed leaching of toxic metal(loid)s using the biological method was more successful than chemical leaching (Deng et al., 2013). The moderate reaction condition, low energy consumption, easy management, lower operational cost, eco-friendliness, minimal environmental effect, and being suited for low-grade mine tailings, residues, and polluted soils makes the bioleaching technique advantageous over the other physicochemical technologies.

These microorganisms used in bioleaching can mobilize toxic metal(loid)s in the soil *via* autotrophic (sulfur and iron oxidizers) and heterotrophic leaching (biosurfactant, siderophore, and dissimilatory reduction; Işildar et al., 2019; Wang et al., 2020). Microorganisms primarily used in bioleaching are summarized in Table 1. Generally, an autotrophic bioleaching process involves microorganisms interacting with metal components through biological and chemical oxidation. The acidophilic microbes involved in autotropic leaching fix carbon dioxide and reenergize it by oxidizing ferrous iron or reduced sulfur. These end products, ferric iron or H_2_SO_4_, formed at the end of this process, which solubilizes metal sulfides and lowers the pH, increasing metal solubilization as microbial leaching is ideal at low pH (1.5–3.0), where most metals dissolve (Sajjad et al., 2019). Besides autotrophic microbes, heterotrophic microbes have also been considered effective in removing toxic metal(loid)s from contaminated soils (Gao et al., 2021) and their mechanism includes acidolysis, complexolysis, and redoxolysis (Desmarais et al., 2020). The ease of adaptation and tolerance of heterotrophic microorganisms have contributed to their effectiveness in bioleaching (Deng et al., 2013). Metal mobilization through heterotrophic bioleaching is achieved by producing and excreting biodegradative organic acids (for example, oxalic, gluconic, malonic, etc.,) which enhance metal solubility through metal chelation (Ren et al., 2009; Wang et al., 2010). This is because the organic acids produced by the heterotrophs render the medium favorable for metal solubilization in the pH range of 4–6, whereas ferric iron is precipitated (Xu et al., 2020). Heterotrophs such as *Pseudomonas aeruginosa* may produce biosurfactants and siderophores to remove toxic metal(loid)s from contaminated soil (Feng et al., 2021).

**Table 1:** A summary of microorganism mostly used in bioleaching.

Microorganism	Metabolism	Optimum Temperature (^ο^C)	Optimum pH	Iron oxidizing	Sulfur oxidizing	Reference
Bacteria						
*Acidiferrobacter thiooxydans*	Autotroph	38	2	+	+	Bulaev et al. (2017)
*Acidimicrobium ferrooxidans*	FA/FM	45–50	∼2.0	+	−	Watkin et al. (2009)
*Acidiphilium cryptum*	FH	35–40	3	+	−	González et al. (2015)
*Acidithiobacillus albertensis*	Autotroph	25–30	3.5–4.0	+	−	Pangayao et al. (2015)
*Acidithiobacillus caldus*	Autotroph	45	2.0–2.5	−	+	Ge et al. (2022)
*Acidithiobacillus ferrooxidans*	Autotroph	28–35	1.4–2.5	+	+	Ko et al. (2013)
*Acidithiobacillus thiooxidans*	Autotroph	Oct-37	2.0–3.0	−	+	Ko et al. (2013)
*Alicyclobacillus disulfidooxidans*	FA/FM	35	1.5–2.5	+	+	Chen et al. (2022)
*Alicyclobacillus GSM*	FA/FM	40–47	1.5–2.0	+	+	Ge et al. (2022)
*Ferrimicrobium acidiphilum*	Heterotroph	< 37	>1.4	+	−	Watling et al. (2014)
*Leptospirillum ferriphilum*	Autotroph	<45	1.0–3.5	+	−	Wang X. et al. (2018)
*Leptospirillum ferrooxidans*	Autotroph	28–30	1.5–3.0	+	−	Nicolova et al. (2017)
*Pseudomonas putida*	Heterotroph	30	7.0–8.0	−	−	Williamson et al. (2021)
*Sulfobacillus benefaciens*	Mixotroph	<47	>0.8	+	+	Nguyen et al. (2021)
*Sulfobacillus thermosulfidooxidans*	Mixotroph	20–60	1.5–5.5	+	+	Zhang et al. (2019)
*Thiomonas cuprina*	Mixotroph	20–45	1.5–7.2	−	+	Wakeman et al. (2011)
Achaea						
*Acidianus brierleyi*	mixotroph	45–75	1.5–2.0	+	+	Dinkla et al. (2009)
*Acidianus infernus*	Autotroph	~90	~2.0	+	+	Panda et al. (2015)
*Acidianus sulfidivorans*	Autotroph	45–83	0.35–3.0	+	+	Panda et al. (2015)
*Acidiplasma cupricumulans*	mixotroph	22–63	0.4–1.8	+	+	Chi et al. (2007)
*Ferroplasma acidiphilum*	mixotroph	15–45	1.3–2.2	+	−	Maulani et al. (2016)
*Metallosphaera hakonensis*	FA/FM	70	3	−	+	Watling et al. (2016)
*Metallosphaera prunae*	FA/FM	~ 75	2.0–3.0	+	+	Ai et al. (2019)
*Metallosphaera sedula*	FA/FM	75	2.0–3.0	+	+	Dinkla et al. (2009)
*Sulfolobus acidocaldarius*	FA	70–75	2.0–3.0	−	−	Börje Lindström et al. (1993)
*Sulfolobus metallicus*	FA/FM	65	2.0–3.0	+	+	Kim et al. (2012)
*Sulfolobus solfataricus*	Mixotroph	85	3.0–4.5	−	−	Roshani et al. (2017)
*Sulfolobus thermosulfidooxidans*	Mixotroph	50–55	1.9–2.4	+	+	Li Q. et al. (2020)
Fungi						
*Aspergillus fumigatus*	Heterotroph	55–70	3.7–7.6			Khan et al. (2019b)
*Aspergillus niger*	Heterotroph	35–37	1.5–9.8			Ren et al. (2009)
*Penicillium chrysogenum*	Heterotroph	35–37	3.5–5.5			Deng et al. (2012)
*Penicillium funiculosum*	Heterotroph	45–55	3.5–4.0			Deng et al. (2013)

FA, Facultative Autotroph; FM, Facultative Mixotroph; FH, Facultative Heterotroph.

Bioleaching considers several physicochemical and biological factors, including the presence of suitable microbial species capable of solubilizing toxic metal(loid)s, the availability of nutrients to stimulate microbial activity, and the ideal pH, temperature, carbon dioxide, and oxygen supply conditions. Therefore, the feasibility of bioleaching to eliminate pollutants in soils requires a comprehensive understanding of the influencing factors. The bioleaching solution’s effectiveness is compromised by the absence of nutrients in it. Therefore, various organic and inorganic nutrients are added to boost bacterial activity in metal-contaminated soils. Bacterial growth and solubilization of toxic metal(loid)s are largely pH-dependent; lower pH is essential for metals to remain stable in solution and for bacteria and archaea that oxidize Fe/S. Optimal growing conditions for microorganisms can lead to high toxic metal(loid)s extraction yields, with oxygen and carbon dioxide concentrations playing a vital role in the growth and activity of the microorganism (Guézennec et al., 2017).

Bioleaching has grown in popularity over the past few decades, attracting several studies. However, a comprehensive review critically examining its optimization and mechanisms for onward improvements in contaminated soils is currently lacking. This paper, therefore, aims to discuss the recent scientific advancement in bioleaching of contaminated soils and the mechanism involved as well as the influencing factors and optimization process. We also present an in-depth review of past and present breakthroughs and the literature gaps in bioleaching processes. The study concludes with the prospects and future considerations of bioleaching for contaminated soils.

## Overview of bioleaching mechanism

In this section, the mechanism of bioleaching of toxic metal(loid)s in contaminated soils has been systematically summarized. Current literature reports indicate that microorganisms involved in bioleaching produce oxidative compounds and sulfuric acids, which aid in solubilizing metals from polluted soils. Additionally, organic acids, siderophores, biosurfactant-producing heterotrophic microbes, and Fe/Mn dissimilatory bacteria have been identified for bioleaching. The bioleaching process is primarily based on the metabolic process and the production of primary products used in oxidation, solubilization, and complexation of toxic metal(loid)s in soil (Johnson, 2018). Improved knowledge of the underlying mechanisms would lead to increased production of primary products of microbes according to the combination of toxic metal(loid)s in the soil and bioleaching process efficiency.

## Autotrophic bioleaching

In general, autotrophs use carbon dioxide from the atmosphere as a carbon source and ferrous iron or elemental sulfur as the main energy source to promote the oxidation of iron and sulfur (Yu et al., 2020). Autotrophs used in leaching include sulfur-oxidizing bacteria (e.g., *Acidithiobacillus thiooxidans*, formerly known as *Thiobacillus thiooxidans, Acidithiobacillus caldus*), iron- and sulfur-oxidizing bacteria (*Acidithiobacillus ferrooxidans, Sulfobacillus benefaciens*), and iron-oxidizing bacteria (*Leptospirillum ferrooxidans*; Nguyen et al., 2021). The genus *Acidithiobacillus* is the most well-studied bacteria in autotrophic bioleaching. They are mostly employed in bioleaching because of their remarkable tolerance to heavy metal toxicity and their requirement of small nutrients for metal mobilization (Roy et al., 2021). Metal sulfides are solubilized by reason of sulfur and iron oxidation by these bacteria, leading to pH reduction and enhancement of the solubilization of additional metal compounds. The energy source for *A. ferrooxidans* comes from the oxidized elemental sulfur, thiosulfate, and ferrous ions, which produce sulfuric acids and ferric ions for metal solubilization. *Acidithiobacillus thiooxidans*, previously *Thiobacillus thiooxidans*, is well-known for its ability to rapidly oxidize elemental sulfur due to its generation of inorganic acids (sulfuric acids). *A. thiooxidans* has the same morphology as *A. ferrooxidans*, but it is distinguished by its significant rapid oxidation of elemental sulfur and its ineptitude to oxidize ferrous ions.

## Mechanism of autotrophic bioleaching

**Figure 1:**
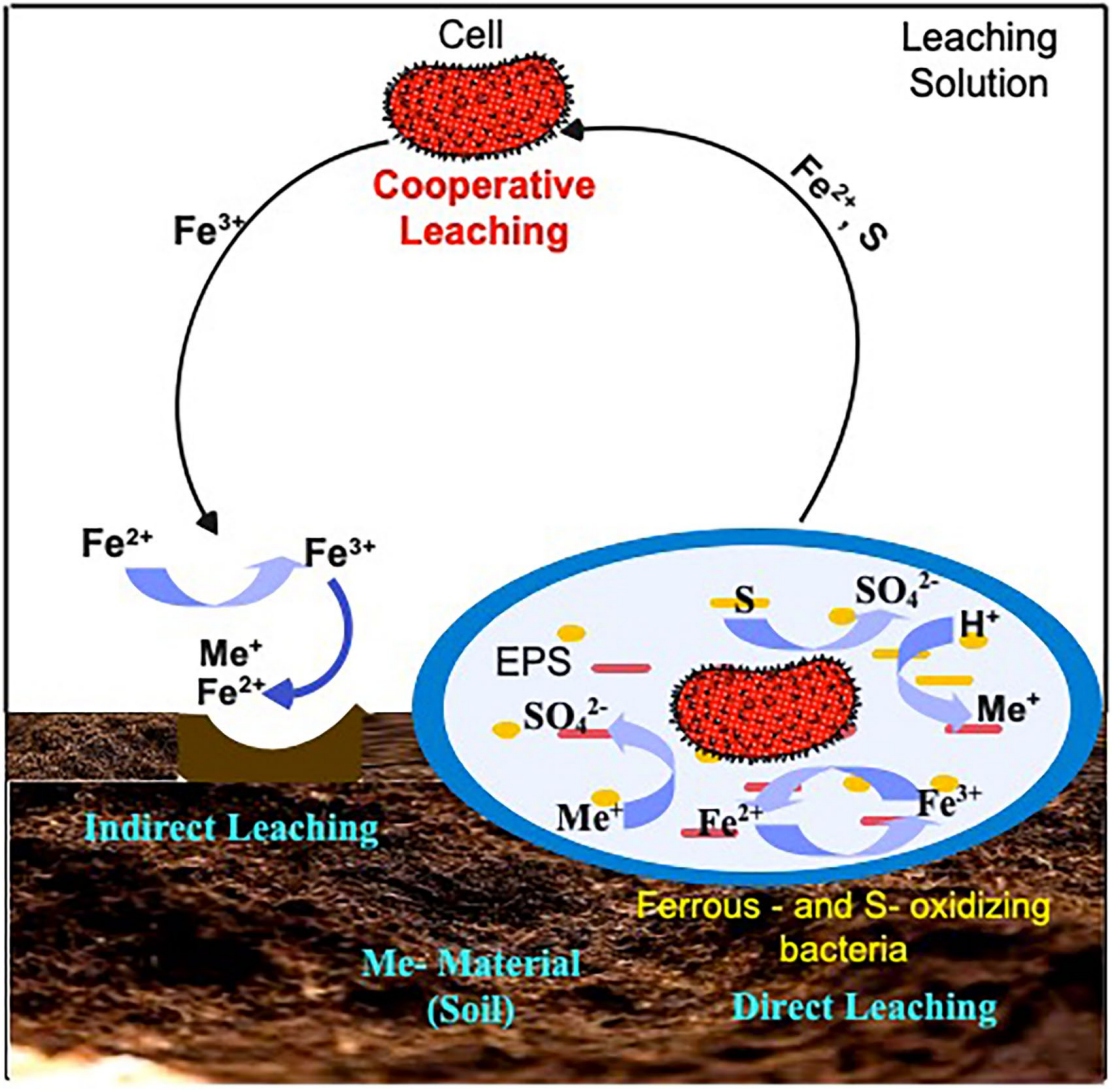
Schematic representation of the autotrophic leaching mechanisms: Contact (Direct): microorganisms oxidizing metal sulfides using a variety of enzymatically catalyzed processes; Non-contact (Indirect): the use of Fe^3+^ to cause oxidation of metal sulfides and reduced to Fe^2+^ and Cooperative leaching: Sulfur and heavy metals undergoing dissolution by means of planktonic cells.

Direct (Contact) bioleaching (including one-step or two-step methods) and indirect (Non-contact) bioleaching (spent-medium bioleaching) processes are two types of autotrophic bioleaching mechanisms (Figure 1). Acidophiles function (in both contact and non-contact mechanisms) by oxidizing ferrous iron (Fe^2+^) into ferric iron (Fe^3+^) and by reducing sulfur to sulfuric acid (H_2_SO_4_; Barnett et al., 2018; Gavrilescu, 2022). In the direct leaching mechanism (Equation 1), microorganisms adhere to sulfide surface and oxidize it enzymatically to soluble metal sulfate. The indirect mechanism requires elemental sulfur or reduced sulfur compounds to be oxidized to sulfuric acid (Equation 2) and a subsequent reduction in pH for promoting metal solubilization (Equation 3; Bosecker, 1997). In this process, Fe^2+^ and reduced sulfur species form in the solution, serving as planktonic cell substrates. Planktonic cells oxidize Fe^2+^ and sulfur species solution to Fe^3+^ and sulfuric acid, respectively. Additionally, Fe acts as an electron carrier, eliminating direct contact for Fe to be oxidized. As the name suggests, “non-contact” or “indirect” mechanism occurs when bacteria are not directly in contact with mineral surfaces. The only function of bacteria is to accelerate the reoxidation of Fe^2+^, which would otherwise take place very slowly without them (Rastegar et al., 2016; Huang et al., 2019; Wu et al., 2019). From the foregoing, it is evident that each of the mechanisms has its benefits and drawbacks. For instance, the toxicity of the recovered metals and other dangerous chemicals may limit microbial growth and metabolic activity in direct bioleaching. Moreover, microorganisms may improve leaching efficiency by (a) adhering to the surface of the soil medium, reducing mass-transfer limitations by producing biolixiviants (e.g., organic acids, chelators) directly on the surface of the metals, (b) metal uptake, adsorption, or complexation by excreted chemicals causing changes in the culture’s equilibrium (Hassanien et al., 2014; Thompson et al., 2018). Nonetheless, there is still a paucity of experimental data to explain how microorganisms disrupt the metal sulfides during contact leaching. In contrast, indirect bioleaching permits microbial leaching agent generation to be optimized separately. It’s worth noting that bacteria contribute to mineral dissolution in both contact and non-contact mechanism by producing the oxidizing agent, the Fe (III) ion, and then oxidizing the sulfur compounds produced by the dissolution. When both direct and indirect bioleaching occurs, a new mechanism called cooperative bioleaching explains this mechanism (Zhao et al., 2015). While this new mechanism is useful for describing the physical state of cells in bioleaching, it does not explain how metal sulfides dissolve within cells. Therefore, autotrophic bioleaching relies on microorganisms and metal compounds interacting biologically and chemically.


(1) $$\mathrm{MeS}+2O_{2}\overset{\mathrm{bacteria}\;}{\to }\;{\mathrm{Me}}^{2+}+{\mathrm{SO}}_{4}$$

(2) $$\mathrm{MeS}+{\mathrm{4Fe}}_{2}{\left({\mathrm{SO}}_{4}\right)}_{3}+{\mathrm{4H}}_{2}O\;\overset{\mathrm{bacteria}\;}{\to }{\mathrm{8FeSO}}_{4}+{\mathrm{MeSO}}_{4}+H_{2}{\mathrm{SO}}_{4}$$

(3) $$2{\mathrm{FeSO}}_{4}+O_{2}+H_{2}{\mathrm{SO}}_{4}\overset{\mathrm{bacteria}\;}{\to }\;\mathrm{Fe}\;{\left({\mathrm{SO}}_{4}\right)}_{3}+H_{2}O$$

(4) $$2H^{+}+\mathrm{Soil}-\mathrm{Me}\to \mathrm{Soil}-2H+{\mathrm{Me}}^{2+}$$

Where Me is a bivalent metal.

Although autotrophic bioleaching is inexpensive, it is occasionally ineffective for specific metals or elements, such as lead and arsenic. Heterotrophic bioleaching, which employs organic materials like yeast extract or glucose as energy and carbon sources, has been developed to address the aforementioned drawback.

## Heterotrophic bioleaching

Heterotrophic bioleaching of toxic metal(loid)s in contaminated soils is accomplished by an indirect process involving the production of organic acids, biosurfactants, and other metabolites that solubilize metal ions (Ren et al., 2009). Some secreted organic acids include oxalic, gluconic, lactic, acetic, succinic, pyruvic, malonic, isocitric, and formic (Jiang et al., 2012). These organic acids produced by heterotrophic microorganisms are crucial for the solubilization of metal ions in soils, given their ability to remove metals from solid matrices by electron transfer and maintain the low pH necessary for efficient bioleaching (Ghosh and Paul, 2017). Siderophores with carbonyl structures play a key role in supplying iron in media with low levels of accessible Fe(III) due to their strong affinity for Fe(III) and Mn(II). They can also chelate these metals using organic acids. Aside from dissolving metal ions from minerals, these metabolites also form soluble metal complexes and chelates that dissolve metal ions from the soil (Williamson et al., 2021; Yi et al., 2022). The fungi species *Aspergillus niger* (*A. niger*) and *Penicillium simplicissimum* are the most commonly utilized heterotrophs for bioleaching due to their ability to excrete large quantities of organic acids such as citric acid, lactic acid, gluconic acid, oxalic acid and siderophore (Table 2; Chaerun et al., 2017; Muddanna and Baral, 2019).

**Table 2:** Organic acids secretion and concentration by microorganism and their leaching efficiency.

Microorganism	Secreted organic acid and concentration	Leaching efficiency	Reference
*Aspergillus tubingensus*	Gluconic acid (265 mM)	Cd 58%, Co 53% & Ni 52%	Din et al. (2020)
	Oxalic acid (24 mM)		
	Fumaric acid (0.34 mM)		
*Aspergillus niger*	Gluconic acid (13,667 mg/L)	One-step bioleaching	Ren et al. (2009)
	Citric acid (6,089 mg/L)	97.5% Cu, 88.2% Cd, 26% Pb & 14.5% Zn	
	Oxalic acid (2,393 mg/L)		
	Malic acid (456 mg/L)		
		Two- step bioleaching	
		56% Cu, 100% Cd, 30% Pb, 19% Zn	
*Aspergillus niger* F2	Glucose as carbon source	One- step bioleaching	Deng et al. (2019)
	Citric acid (27.581 mg/L)	55.19% Cd, 72.3% Cu, 100% Pb, 100% Zn	
	Malic acid(31.152 mg/L)		
	Butanedioic acid (49.616 mg/L)		
		Two- step bioleaching	
	Sucrose as carbon source		
	Citric acid (174.262 mg/L)	52.8% Cd, 73% Cu, 69.8% Pb, 97.3% Zn	
	Malic acid (7.047 mg/L)		
	Butanedioic acid (45.528 mg/L)		
	Pyroracemic acid (1.833 mg/L)		
*Penicillium chrysogenum* strain F1	One -step bioleaching	One step bioleaching	Deng et al. (2012)
	472.6 mg/L of Glucose acid, Oxalic acid and pyruvic acid	50% Cd, 35% Cu, 9% Pb, 40% Zn	
	Two – step bioleaching	Two- step bioleaching	
	520.8 mg/L of glucose acid, oxalic acid and pyruvic acid	63% Cd, 56% Cu, 14% Pb, 54% Zn	
*Aspergillus niger* Strain SY1	One- step bioleaching	One step bioleaching	Zeng et al. (2015)
	Oxalate (6048.7 mg/L)	93.5% Cd, 62.3% Cu, 68.2% Zn	
	Citric acid (367.5 mg/L)		
	Gluconic acid (1616.4 mg/L)		
	Pyruvic acid (10.225 mg/L)	Two – step bioleaching	
	Succinic acid (2.085 mg/L)	99.5% Cd, 71.9% Cu, 76.4% Zn	
	Lactic acid (0.266 mg/L)		
	Acetic acid (0.234 mg/L)		
	Two – step bioleaching		
	Oxalate (5751.3 mg/L)		
	Citric acid (260.2 mg/L)		
	Gluconic acid (3035.1 mg/L)		
	Pyruvic acid (3.664 mg/L)		
	Succinic acid (3.914 mg/L)		
	Lactic acid (0.188 mg/L)		
	Acetic acid (0.184 mg/L)		

## Mechanism of heterotrophic bioleaching

**Figure 2:**
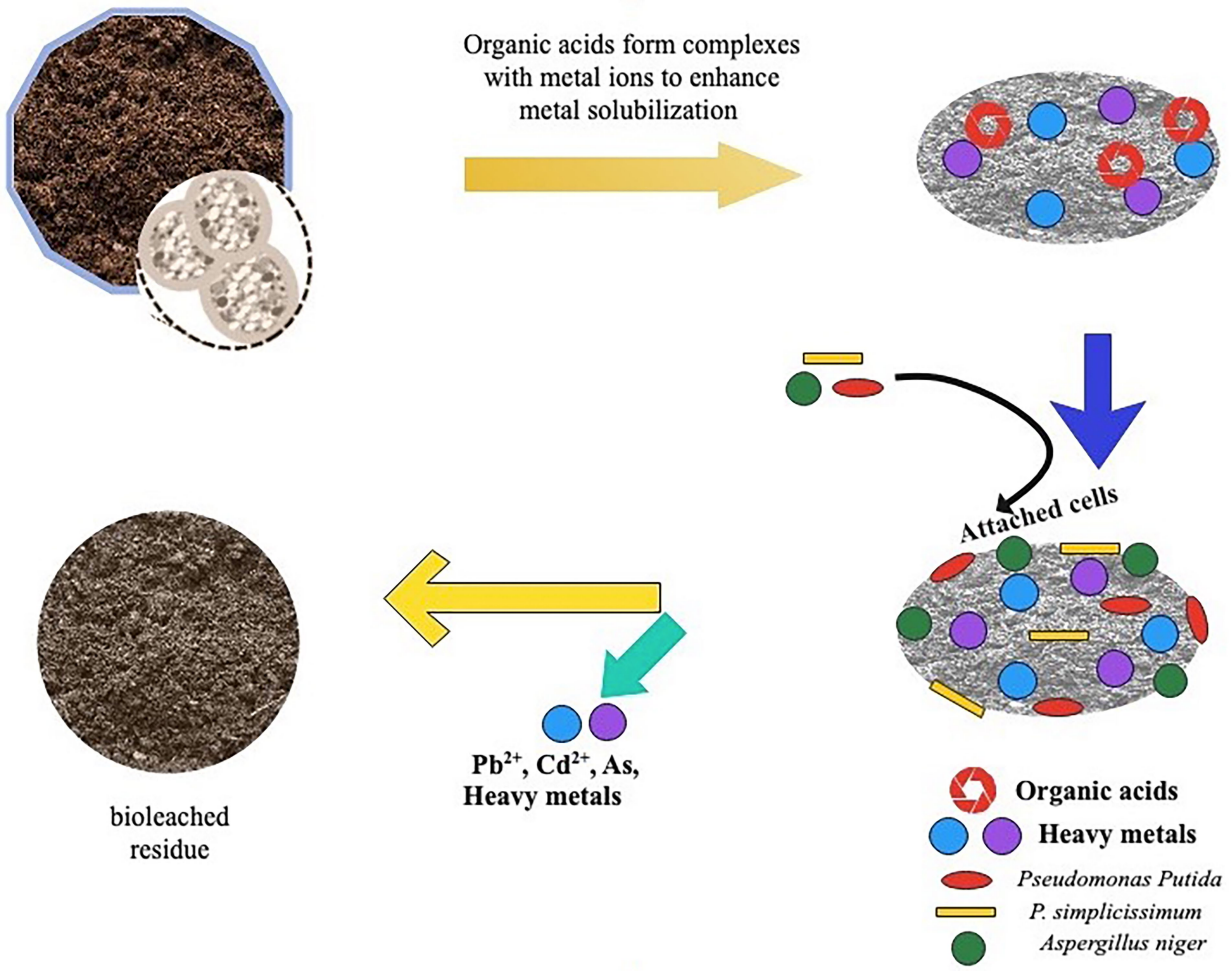
Schematic diagram of toxic metal(loid)s solubilization in contaminated soil by organic acids. Organic acids produced by bacteria facilitate solubilization by supplying protons and metal complexing organic acids anions. This results in ion exchange, proton -promoted mechanism releasing toxic metal (loid)s from contaminated soil.

Organic acids primarily use two mechanisms to mobilize and leach metals from soil: acidolysis and complexolysis (Figure 2). Acidolysis is an indirect leaching process in which oxygen atoms coating an insoluble metal compound are protonated and made soluble by organic acid (Glombitza and Reichel, 2013). In acidolysis, also referred to as proton-induced metal solubilization, microbes release protons (H^+^) from the mineral surface, which bind and release metal ions (Cornu et al., 2017). As a result of the protonation of the anion, the metal is detached from the soil and solubilized. This explains why most metals in the soil become more mobile as pH decreases. Furthermore, the H^+^ produced by the organic acids stabilizes the metal chelation while increasing the mobilization of metal ions. The phenomenon behind this is that, as protons are generated during the dissociation of acids, more potent acids will likely produce a greater quantity of H^+^ in the leaching medium. For this reason, the strength of organic acid is essential in selecting the right organic acid for metal leaching (Pathak et al., 2021). Acidolysis is the rapid and common leaching mechanism for fungi and other heterotrophic organisms such as *Acidithiobacillus ferrooxidans* and *Leptospirillum ferrooxidans.* The chelation mechanism is used to achieve complexolysis, which balances the metal ions in the solution by acidolysis (Srichandan et al., 2019; Dusengemungu et al., 2021). Complexolysis, also known as ligand-induced metal solubilization, occurs when metals and microbial chelators are combined at the surface of metal-bearing phases, allowing the metal to be released from the surface and cationic constituents to be withdrawn from the mineral lattice (Brandl and Faramarzi, 2006; Cornu et al., 2017; Esmaeili et al., 2022). For instance, oxalic acid forms a complex with Al, Fe, and Mg or a complex of citric acid with Ca and Mg. The leaching rate is influenced by the organic acid’s complexity and the complex’s stability. This indicates that the leaching efficiency increases significantly with the strength of the ligand and the difficulty of its retention or adsorption by the soil (Pathak et al., 2021). Examples of heterotrophic bacteria and fungi involved in complexolysis include; *Chromobacterium violaceum, Pseudomonas aeruginosa*, *Pseudomonas flourescens*, and *Bascillus megaterium.*

**Figure 3:**
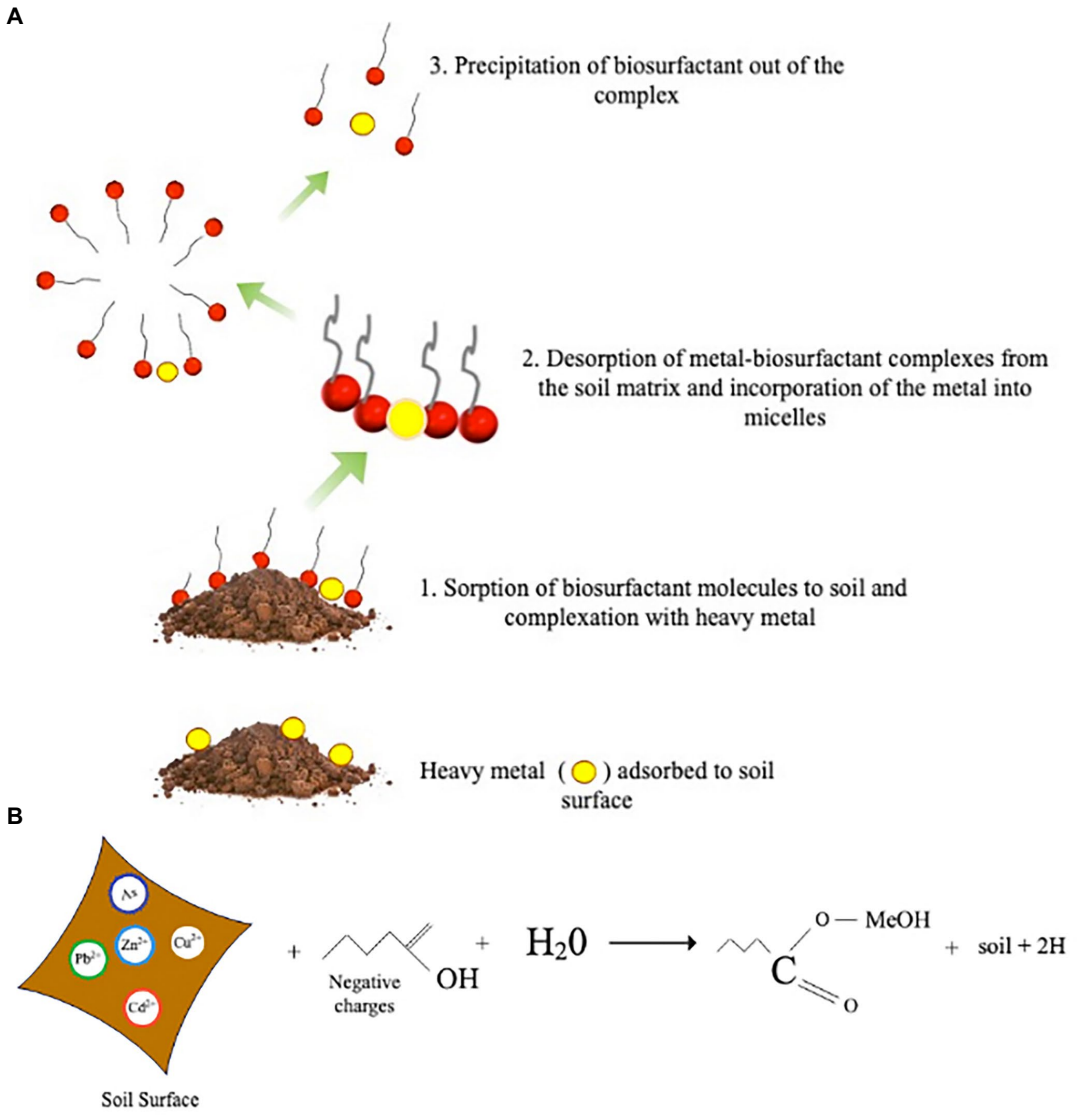
**(A)** Mechanism of heavy metal removal from contaminated soil by the action of microbial biosurfactants occurs in the following order (1) sorption of the biosurfactant to the soil surface and complexation with the metal (2) desorption of metal from the soil (3) micelle formation and removal of heavy metal *via* precipitation **(B)** Chemical reaction between heavy metal contaminated soil, water and biosurfactant.

Heterotrophic bioleaching also involves using biosurfactants to solubilize toxic metal(loid)s from contaminated soils. Biosurfactants are readily accessible surface-active compounds primarily produced by bacteria, fungi, and plant metabolites (Akbari et al., 2018). Biosurfactants’ hydrophobic and hydrophilic properties make them good complexing agents suitable for heterotrophic bioleaching (Luna et al., 2016). Furthermore, because biosurfactant contains more functional groups and has a larger molecular structure, it has an unusual surface activity that facilitates toxic metal(loid)s extraction (Tang et al., 2017). Figure 3A illustrates the mechanism of toxic metal(loid) removal from polluted soil utilizing biosurfactants. Ion exchange, precipitation-dissolution, and counter-ion association are all plausible mechanisms for toxic metal(loid)s removal by biosurfactants (Açikel, 2011). Three primary steps are involved in removing toxic metal(loid)s from the soil through bioleaching with biosurfactant. In an aqueous solution, toxic metal(loid)s adsorbed on soil particles detach due to the sorption of biosurfactant molecules at the interfaces between the soil and the metal. The most important mechanism is the microbial accumulation of metal ions under limited interfacial activity by the direct contact of the biosurfactant on the interface of the solid-solution phases (Mulligan et al., 1999). For example, toxic metal(loid)s are removed from contaminated soils by forming complexes with biosurfactants on the soil surface and are leached from the soil into the solution as the interfacial tension is reduced.

Another mechanism is the complexation of free forms of metal residing in solution. Le Chatelier’s principle explains this as reducing the metal’s solution phase activity to facilitate desorption (Das et al., 2017). The biosurfactants structural size and charge also impact the mobility of biosurfactant–metal complexes *via* the soil (Usman et al., 2016). Anionic biosurfactants have a significant chelating affinity for positively charged heavy metal ions, such as Ni^2+^, Zn^2+^, and Cu^2+^, due to their complexation capacity, whereas cationic biosurfactants have a complexation affinity for negatively charged ions (Figure 3B; Müller et al., 2012; Mulligan, 2021). The electrostatic attraction might account for the significant chelating affinity. Additionally, biosurfactants are crucial in eliminating toxic metal(loid)s from the soil by promoting micelle formation through its limited interfacial activity and surface tension (Sarubbo et al., 2015). The biosurfactants absorb the metals and trap them within the micelle using electrostatic interactions. Also, the solubility and mobility of toxic metal(loid)s are increased by micelles that bind to oppositely charged metal ions, enhancing toxic metal(loid)s recovery (Mishra et al., 2021). The mechanism of micelle production and critical micelle concentration (CMC) has a role in solubilization and mobilization (Costa et al., 2010; Das et al., 2017). Solubilization increases as a result of micelle formation and surface tension reduction. Consequently, biosurfactants can effectively solubilize toxic metal(loid)s from contaminated soils.

**Figure 4:**
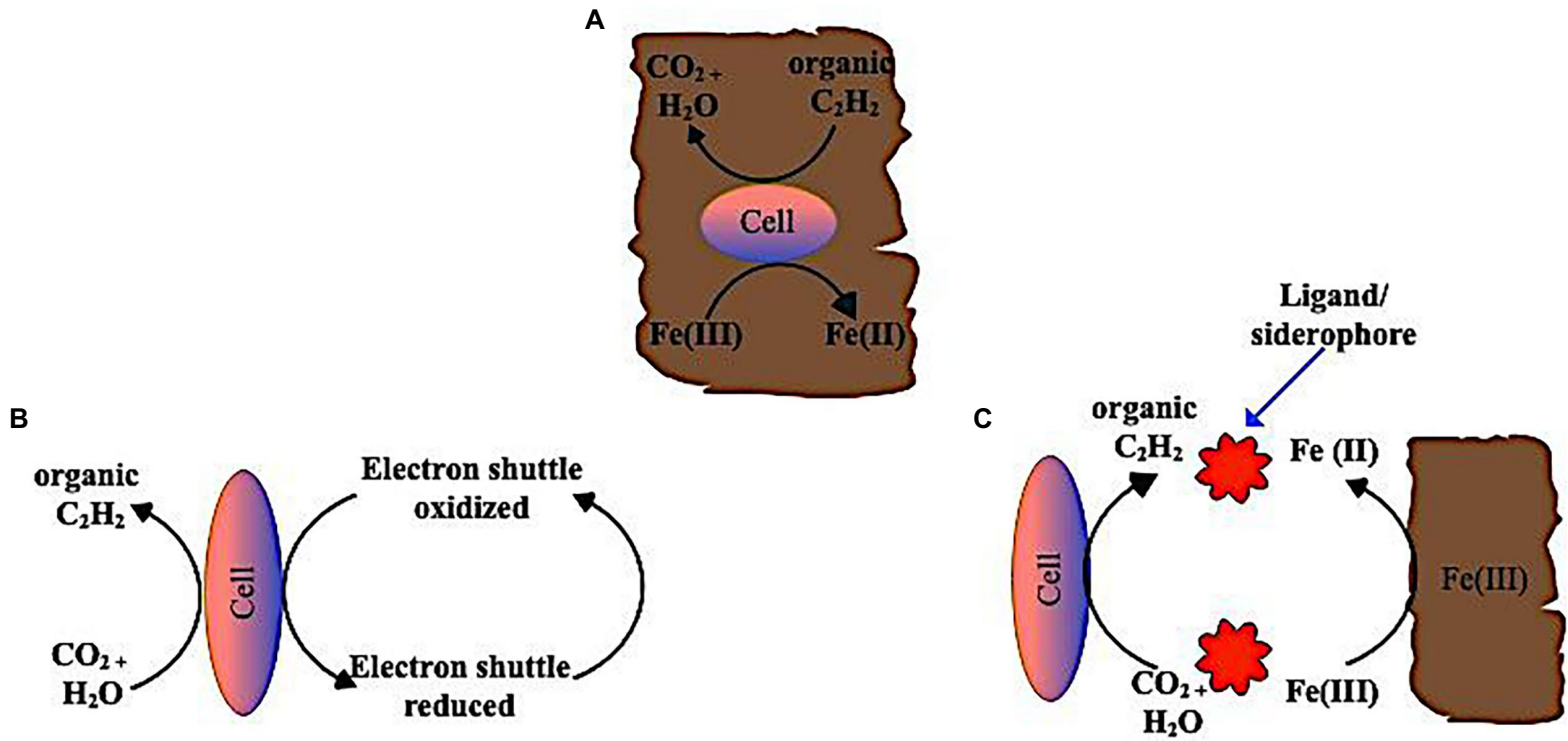
Mechanism of dissimilatory reduction of insoluble Fe (III) oxides in soil *via* **(A)** direct contact of bacteria cells to oxide surface of Fe(III) **(B)** extracellular electron shuttle mediating transfer of solid phase Fe(III) oxides and bacteria cells **(C)** Ligands (chelators/siderophores) helps solubilize solid phase Fe(III) and make Fe(III) in soluble form readily available to the bacteria cells.

Toxic metal(loid)s are readily absorbed by Fe(III) and Mn(IV) oxides, which may make their removal from contaminated soils difficult. Therefore, introducing humic materials or similar compounds may speed up the process of microbial reduction of Fe(III) and Mn(IV), which might be one method to extract these metals (Aishvarya et al., 2019). Microbes capable of reducing Fe(III) can also reduce Mn(IV) and vice versa. Firmicutes, Proteobacteria, Deferribacteres, Thermotogae, and Actinobacteria form the majority of these microbes (Lovley, 1991; Esther et al., 2015). Generally, these organisms are described as dissimilatory Fe(III) or Mn(IV) reducing microorganisms based on their accumulation of Fe(II) or Mn(II) under anaerobic conditions in organically complex media (Lloyd, 2003). The most extensively researched genera of metal-reducing bacteria are *Shewanella* and *Geobacter,* and it has been established that they are capable of reducing insoluble Fe(III) and Mn(IV) oxides (Richter et al., 2012; Novotnik et al., 2019). Several Fe(III) oxides, including α-Fe_2_O_3_ (hematite), γ-Fe_2_O_3_ (maghemite), α-FeOOH (goethite), γ-FeOOH (lepidocrocite), and Fe(OH)_3_ (amorp) may be found in the soil under aerobic circumstances (Zhang et al., 2012). Fe solubility is very low in well-aerated soils, which is ultimately controlled by the soil’s most insoluble oxide, goethite. Figure 4 illustrates the mechanisms for dissimilatory iron reduction by microorganisms; (i) Direct contact between microorganisms, eg. *Geobacter* spp. and the solid-phase Fe(III) in the soil is described as the mechanism for the reduction of insoluble Fe(III) (Esther et al., 2015). Specific bacteria, such as *Geobacter metallireducens*, have cell appendages, such as flagella and pili, that prefer Fe (III) compounds and may function as electrical conduits and transfer electrons to insoluble Fe(III) oxides to accomplish an iron reduction in certain circumstances (Reguera et al., 2005). Hydrophobic proteins (also known as nanowires) make up the majority of the flagellum. Previous studies have proven that a flagellate cells are less hydrophobic than flagellated cells (such as *Shewanella*; Qian et al., 2014). Thus, flagella attach to the surface of crystalline Fe (III) (hydro)oxides, acting as an electrical bridge for electron transport. This is contrary to earlier studies that claimed *Geobacter* spp. directly attach to the Fe (III) oxide surface through pili production (Childers et al., 2002). (ii) In addition to the direct contact mechanism, some smaller molecules can operate as ‘electron shuttles’ between dissimilatory iron-reducing bacteria and Fe(III) compounds by oxidizing reductive chemicals released by dissolved organic matter in the soil. This mechanism eliminates the necessity of direct contact between the microbes and the minerals. For example, humics, and other extracellular quinones, are used as electron acceptors by Fe(III)-reducing bacteria, while reduced hydroquinone moieties are used to abiotically transfer electrons to Fe(III) minerals (Lovley et al., 1996, 2004; Von Canstein et al., 2008).

## Application of bioleaching in toxic metal(loid)s contaminated soil

In particular, Cd and As contamination of paddy fields poses a significant risk to rice grain (Meharg and Rahman, 2003; Zhang et al., 2021). For this reason, Cd and As contamination of paddy fields has led to public concern due to their enormous risk to global food safety and human health. To date, bioleaching which is focused on the solubilization of metals with microorganisms, has emerged as one efficient method to deal with this problem (Li D. et al., 2020). The use of heterotrophs in bioleaching has gained significant interest due to the secretion of organic acid. Bioleaching of toxic metal(loid)s in polluted soils involving fungi (*Penicillium* spp. and *Aspergillus* spp.) as well as sulfur, and iron-oxidizing bacteria have been utilized in recent years (Table 3). For example (Ge et al., 2022), investigated the use of a microbial consortium enriched with sulfur-oxidizing bacteria in enhancing Cd and Zn removal from toxic metal(loid)s contaminated paddy soil. Their findings showed the bioleaching group having higher removal effectivity for Cd and Zn than the chemical treatment (acid leaching and sulfur treatment). Removal of Cd from contaminated paddy soils by a consortium of autotrophic and indigenous cadmium-tolerant bacteria has also been investigated in the laboratory (Deng et al., 2017). Findings from the study showed a higher leaching rate of Cd, ranging from 74.93% to 92.76%, and a significant change in the distribution of Cd fractions before and after bioleaching, with an organic fraction and residues fraction remaining the most stable was observed. Tran et al. (2020) also in a laboratory-scale experiment reported on the impact of an indigenous microbial consortium on the removal of As from As-contaminated agricultural soil utilizing *Shewanella putrefaciens*, a Fe(III)-reducing bacterium. Their investigation found that using *S. putrefaciens* with the indigenous bacterial consortium resulted in the best As removal effectiveness (57.5%). The indigenous bacteria and *S. putrefaciens* alone removed 16.4% and 30.1% of As from the soil, respectively.

**Table 3:** The use of bioleaching for treatment of various types of toxic metal(loid)s polluted soil.

Soil type	Pollutant	Bioleaching type	Bioleaching Microorganisms	Scale	Country	Major observations	References
Paddy soil	Cd, Zn	Autotrophic	A sulfur- oxidizing microbial consortium (*A. caldus* S2, *S. thermosulfidooxidans* YN22, *Alicyclobacillus* Bio504 and *S. acidophilus* TPY)	Lab. scale	China	The highest removal efficiency was observed in the bioleaching with the microbial consortium compared to acid leaching and sulfur treatment	Ge et al. (2022)
Paddy soil	Cd	Autotrophic	Consortium of autotrophic and indigenous cadmium-tolerant bacteria	Lab. Scale	China	Leaching rate of Cd ranged from 74.93% to 92.76%	Deng et al. (2017)
Contaminated Agricultural soil	As	Heterotrophic (Fe dissimilatory reduction)	*Shewanella putrefaciens*	Lab. Scale	South Korea	57% As was removed with *S. putrefaciens* and indigenous bacterial consortium coexisting in the soil	Tran et al. (2020)
Contaminated soil with smelting slag	Cd, Cu, Pb, Zn, Mn, Cr	Heterotrophic (organic acids)	*Penicillium* *chrysogenum* *strain* F1	Lab. Scale	China	Removal efficiency of Cd 74%, Cu 59%, Pb 24%, Zn 55%, Mn 57 and Cr 25% were higher than chemical leaching	Deng et al. (2013)
Contaminated Sandy Soil	Cd, Pb, Zn, Cu	Heterotrophic (organic acids)	*Aspergillus niger*	Lab. Scale	China	Results after One-step process showed that maximum removal of 56% Cu, 100% Cd, 35% Pb, 19% Zn was achieved	Ren et al. (2009)
						After Two-step process highest removal efficiencies of 97.5% Cu, 88.2% Cd, 26.6% Pb and 14.5% Zn was also achieved	
Contaminated soil from smelting site	Cd, Pb, Zn	Heterotrophic (organic acids)	*Aspergillus flavus*	Lab. scale	China	The use of *Aspergillus flavus* in both One-step and two-step bioleaching process resulted in the removal efficiencies of One- step process: 39.77% Cd 18.16% Pb: 58.22% Zn	Qayyum et al. (2019)
						Two- step process 49.66% Cd: 16.91% Pb: 65.73% Zn	
Contaminated Soil	Cd, Cr	Heterotrophic (organic acids)	*Aspergillus fumigatus* (M3Ai), *Aspergillus niger* (M1DGR), *Penicillium rubens* (M2Aiii)	Lab. scale	Pakistan	Two-step Bioleaching using *Aspergillus fumigatus* (M3Ai) resulted in Cd 79% Cr 69% removal efficiencies	Khan et al. (2019a)
						Two-step bioleaching: *Aspergillus niger* (M1DGR) 98% Cd: 43% Cr:	
						Two-step bioleaching: *Penicillium rubens* (M2Aiii) 98% Cd:	
Contaminated soil from Pb-Zn smelting site	As, Cd, Cu, Mn, Pb	Heterotrophic (biosurfactant)	*Burkholderia* sp. Z-90	Lab. Scale	China	Combining bioleaching by the gross biosurfactant of *Burkholderia* sp. *Z-90* resulted in the maximum removal efficiencies of 31.6% As: 37.7% Cd: 24.1% Cu: 52.2% Mn: 32.5% Pb: 44.0% Zn	Yang et al. (2018b)
Artificial Polluted Garden soil	Cd, Cu, Pb	Heterotrophic (biosurfactant)	*Pseudomonas* sp. CQ2,	Lab. Scale	China	Highest removal efficiencies of 78.7% Cd, 65.7% Cu and 56.9% Pb were achieved	Sun et al. (2021)
Paddy soil	Cu, Pb, As	Autotrophic	*Acidithiobacillus thiooxidans*	Lab. Scale	Korea	1% (w/v) sulfur addition led to the removal efficiencies of 67.6% Cu, 25.8% Pb and 53.3% As	Han and Lee (2015)
Contaminated soil from mining site	Fe, Zn	Autotrophic	*Acidithiobacillus thiooxidans* and *Acidithiobacillus ferrooxidans*	Lab. Scale	Spain	Bioleaching alone removed: 50% Zn and 19% Fe. Combing bioleaching with rhamnolipids biosurfactant: 70% Zn and 36% Fe	Diaz et al. (2015)
Contaminated Agricultural soil	Cd	Autotrophic	mixotrophic acidophiles	Lab. Scale	China	Two step bioleaching removed 34% Cd	Hao et al. (2019)
Contaminated soil from Au-Ag mine site	As	Autotrophic	Sulfur oxidizer *Acidithiobacillus thiooxidans* and Iron oxidizer *Acidithiobacillus ferrooxidans*	Lab. Scale	South Korea	Bioleaching had higher leaching efficiency than chemical leaching. *A. ferrooxidans* could remove 70% As while *A. thiooxidans* removed 40% As	Ko et al. (2013)
Artificial contaminated loamy soil	Cr (VI)	Autotrophic	sulfur oxidizing bacteria *Acidithiobacillus thiooxidans*	Lab. scale	Portugal	A maximum removal of 83% was obtained for the soil contaminated with 50 mg kg^−1^of Cr(VI), at pH 2, and 26°C	Fonseca et al. (2014)
Contaminated soil from Industrial area	Cd, Cr, Pb, Zn, Fe, Cu	Autotrophic	sulfur oxidizing bacteria *Acidithiobacillus thiooxidans*	Lab. Scale	India	After bioleaching cadmium, copper and zinc were removed entirely from all the fractions of soil except the residual fraction	Naresh Kumar and Nagendran (2009)
Contaminated soil from smeltery site	Cd, Pb, Cu, Zn	Heterotrophic (organic acids)	*Aspergillus niger* F2	Lab. Scale	China	The bioleaching efficiency was higher when sucrose was used as the carbon source than glucose. The bioleaching ratio for Cd, Pb and Zn was greater with one-step bioleaching except for Cu	Deng et al. (2019)
Chromium slag contaminated site soil	Cr	Heterotrophic	*Geotrichum* sp. G1 and *Bacillus* sp. B2	Lab. Scale	China	The mixed culture of *Geotrichum* sp. G1 and *Bacillus* sp. B2 resulted in 94.8% Cr(VI) reduction efficiency	Qu et al. (2018)
Contaminated soil from Pb-Zn Smelting site	Pb, Zn, Cr, Cd, Cu, Mn		*Providencia* sp. LLDRA6	Lab. Scale	China	Combining *Providencia* sp. LLDRA6 BioMnOx exhibited significant removal efficiency of Pb (81.72%), Cr (88.29%), Cd (90.34%), Cu (91.25%), Mn (56.13%), and Zn (59.83%) from contaminated soils	Li D. et al. (2020)
Forest Soil	Cd, Cu, Zn	Autotrophic	*Acidithiobacillus ferrooxidans, Acidithiobacillus thiooxidans* and *Leptospirillum ferrooxidans*	Lab. Scale	Bulgaria	After a leaching period of 170 days, the toxic metal(loids) concentration significantly decreased below the appropriate permissible levels	Nicolova et al. (2017)
Contaminated soil from Industrial area	Pb(II), Cr(VI)	Heterotrophic (organic acids)	*Aspergillus flavus* (F3)	Lab. Scale	China	Bioleaching exhibited greater removal efficiency of 36% Pb(II) and 99% Cr(VI) than chemical leaching 21.30% Pb (II) and 72% Cr(VI)	Qayyum et al. (2016)

The anthropogenic activities such as industrialization, smelting, and mining have resulted in numerous contaminated soil (Bolan et al., 2014; Yang et al., 2018a). Although the use of heterotrophic microorganisms for bioleaching of contaminated soil is still in its infancy, its effectiveness in extracting toxic metal(loid)s from contaminated soils cannot be underestimated. Among bacteria, the genus *Bacillus spp*. and *Pseudomonas* spp. are highly effective for metal solubilization. Regarding fungi, the genera *Aspergillus* and *Penicillium* are the most important ones for bioleaching (Rasoulnia et al., 2016; Vakilchap et al., 2016). For instance, Deng et al. (2013) researched the bioleaching mechanism of toxic metal(loid)s from contaminated soil using indigenous *Penicillium chrysogenum* strain F1. Results indicated that bioleaching had greater removal efficiencies than chemical leaching, with Cd, Cu, Pb, Zn, Mn, and Cr removal percentages reaching 74%, 59%, 24%, 55%, 57%, and 25%, respectively. Derived organic acids from *Aspergillus niger* have also shown more significant tendencies for leaching toxic metal(loid)s from contaminated soil in an industrial area (Ren et al., 2009). Speciation of toxic metal(loid)s chemical forms using a one-step and two-step approach showed the one-step bioleaching process exhibiting a higher removal efficiency of 56% Cu, 100% Cd, 30% Pb, and 19% Zn than the two-step bioleaching process of 97.5% Cu, 88.2% Cd, 26% Pb, and 14.5% Zn (Ren et al., 2009). In contrast to works by Ren et al. (2009) and Qayyum et al. (2019), in two-step bioleaching with *Aspergillus flavus,* revealed that the *Aspergillus. flavus* significantly removed 49.66% Cd and 65.73% Zn, more than one-step bioleaching of 39.77% Cd and 58.22% Zn (Qayyum et al., 2019). Khan et al. (2019b) also isolated and identified indigenous fungi strains from contaminated soil and studied their potential use for toxic metal(loid)s removal from the contaminated soil. Among the three metallotolerant fungal strains isolated for bioleaching, *Aspergillus niger* (M1DGR) was found to be successful for Cd removal, as it effectively removed 98% Cd from the contaminated soil. Furthermore, *Aspergillus fumigatus* strain (M3Ai) could effectively remove 79% Cd and 69% Cr from the contaminated soil, making both strains (M3Ai & M1DGR) potential candidates for remediating soil contaminated with Cd and Cr (Khan et al., 2019a). A novel native bacterium *Providencia* sp. LLDRA6 in combination with BioMnO_X_ wash highly effective in removing 81.72% Pb, 88.29% Cr, 90.34% Cd, 91.25% Cu, 56.13% Mn, and 59.83% Zn as compared to the *Providencia* sp. LLDRA6 alone (Li D. et al., 2020). Despite the lower leaching efficiency of bacteria alone, they facilitated the transformation of metal residues into easily migratory fractions. Bioleaching by biosurfactants has also received considerable attention over the last few decades, and this could be attributed to them not producing any secondary pollutants (Mao et al., 2015; Kaczorek et al., 2018). Toxic metal(loids) are removed from soils by bioleaching using biosurfactants by chelating metals with their functional groups as well as increasing their mobility by changing the metals’ speciation fractions.

*Burkholderia* sp. Z-90 (biosurfactant producer) isolated from a cafeteria sewer sludge was also successfully used to remove toxic metal(loids) from contaminated soil (Yang et al., 2016). Again, the biosurfactant and glycolipids producer *Burkholderia* sp.Z-90 used with poly aluminum chloride flocculation significantly enhanced bioleaching efficiency (Yang et al., 2018b). Biosurfactant derived from *Pseudomonas* sp. CQ2 and its underlying mechanism for removing toxic metal(loid)s from contaminated soil have also been investigated (Sun et al., 2021). The removal efficiencies of Cd, Cu, and Pb could reach up to 78.7%, 65.7%, and 56.9%, respectively. Furthermore, ATR-FTIR revealed that biosurfactant carboxyl functional groups form complexes with Cd, Cu, and Pb, and this explains how *Pseudomonas* sp. CQ2 biosurfactant could efficiently remove toxic metal(loid)s from the soil (Sun et al., 2021). Toxic metal(loid)s are more likely to be mobilized and accumulate in an acid-to-neutral pH range. For example, the ionization of the carboxylic functional group of rhamnolipids (COO-) is favored by acidic pH, therefore allowing metal cations to bind more strongly to the biosurfactant. Moreover, increasing the pH of the biosurfactant solution can lower the interfacial tension. In a study by (Wu L. et al., 2020), Pb removal from soil using rhamnolipids was enhanced by increasing the pH from 4.0 to 7.0, which decreased the adsorption of rhamnolipids on soil and facilitated the desorption of heavy metals *via* biosurfactant–metal complexation. A laboratory-scale experiment on the bioleaching of paddy soil contaminated with Cu, Pb, and As using sulfur-oxidizing bacterial *Acidithiobacillus thiooxidans* have been studied (Han and Lee, 2015). Various sulfur concentrations (0%, 0.1%, 0.5%, and 1%) were used in batch studies to determine the removal effectiveness of toxic metal(loid)s. The results showed that sulfur enhanced the removal of toxic metal(loid)s. Again, the results demonstrated that adding sulfur was essential in the extraction process for bacteria metabolism to function effectively; nevertheless, Pb removal efficiency was relatively low, which can be attributed to the formation of insoluble PbSO_4_ precipitates (Han and Lee, 2015). The feasibility of the bioleaching technique using sulfur-oxidizing bacteria *Acidithiobacillus thiooxidans* for the treatment of heavy metal-contaminated shooting range soil has also been explored (Han et al., 2009). The leaching efficiency was significantly influenced by sulfur concentration, operating temperature, and the amount of bacteria inoculum. This was evident in their results which showed increased sulfur concentration and bacteria inoculum coupled with an operating temperature of 26°C ensured higher extraction of heavy metals from the contaminated soil. Additionally, the influence of initial pH on bioleaching systems for removing toxic metal(loid)s from contaminated soil utilizing *Acidithiobacillus thiooxidans* has also been studied (Kumar and Nagendran, 2007). At varied initial pH, the solubilization of chromium, zinc, copper, lead, and cadmium ranged from 59% to 98%. However, increasing the pH of the bioleaching system toward neutral had no effect on *Acidithiobacillus thiooxidans* ability to use elemental sulfur. Furthermore, several studies have examined mixing autotrophic and heterotrophic microorganisms to improve metal extraction efficiency (Yang et al., 2018b; Hao et al., 2019; Xu et al., 2020). In addition, previous studies showed that mixotrophic acidophiles with both autotrophic and heterotrophic species are more capable of performing complex tasks in natural soils due to their better environmental adaptability than pure species (Kang et al., 2016; Latorre et al., 2016). Extraction of Fe and Zn was enhanced in a mixed culture of *A. thiooxidans* and *A. ferrooxidans* with a biosurfactant-producer *Pseudomonas aeruginosa* CVCM411(Diaz et al., 2015). In a two-step bioleaching approach, a mixotrophic acidophilic consortium extracted 34% total Cd and 87% available Cd, which was higher as compared to acid treatment of 12% of total Cd and 51% of available Cd (Hao et al., 2019). In addition, the mixotrophic acidophilic consortia increased metal absorption into plant tissues by 78% from the treated soils, improving the Cd removal efficiency. Intriguingly, the mixotrophic acidophiles did not colonize soils; rather, they did induce an increase in the composition of indigenous bacteria such as *Alicyclobacillus*, *Clostridium sensu strict*, and *Streptacidiphilus*. In summary, the above discussion suggests that autotrophic and heterotrophic microbes can be utilized to leach toxic metal(loid)s from contaminated soil. In heterotrophic bioleaching, *Aspergillus niger* is primarily used because it produces high organic acids needed for metal solubilization. Similarly, autotrophic and heterotrophic microbes vary in their bioleaching ability with variations in operational parameters; hence maximum bioleaching occurs under optimal conditions for bacteria growth.

## Influencing factors affecting the bioleaching process

**Figure 5:**
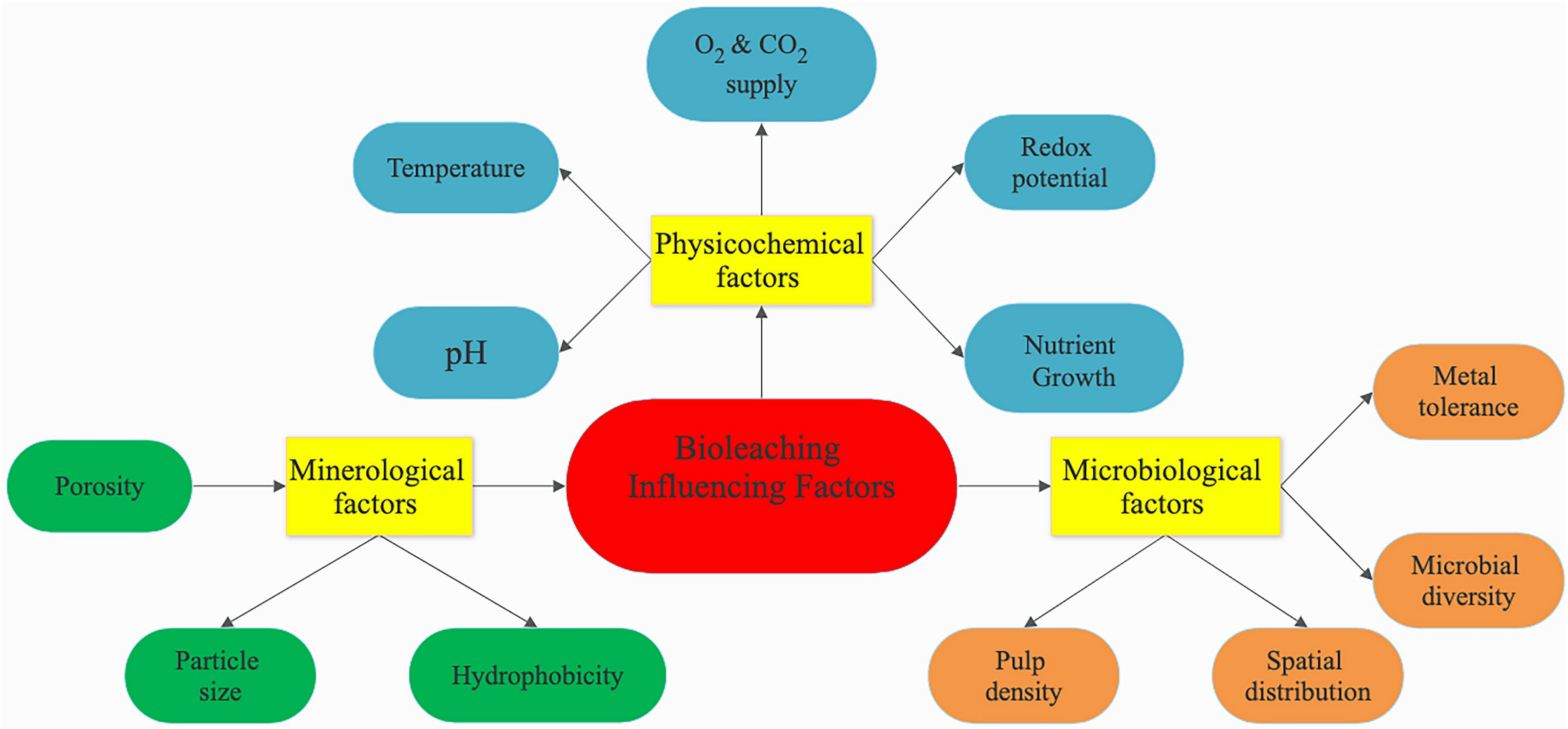
Influencing factors affecting the bioleaching process.

The bioleaching process has several factors (physiochemical, microbiological, or mineralogical) that can influence bioleaching (Figure 5). These factors can affect the bioleaching process individually or in some cases, a combined effect of one or two factors can influence the leaching efficiency. Therefore, various operational parameters must be well adjusted to increase bioleaching performance. For example, autotrophic bacteria need an acidic pH to leach metals, whereas heterotrophic bacteria need a relatively high pH to solubilize metals. The leaching efficiency is enhanced by optimizing the growth conditions of the microbes as well as improving the synthesis of the essential metabolites. The physiochemistry factors that must work harmoniously to ensure optimal bioleaching include pH, redox potential, temperature, oxygen, carbon dioxide supply, pulp density, and growth nutrient. Microbiological factors include microbial diversity, which refers to the diversity of microorganisms found in a conducive environment for bioleaching, such as bacteria, fungi, algae, flagellates, and those found in microbial biocenosis, as well as microbial community diversity, metal tolerance, and spatial distribution. The type of the processed mineral and characteristics like particle size that impact dissolution rate, porosity, and hydrophobicity are all considered mineralogical factors (Cheru, 2021).

## pH

pH is of significant importance in bioleaching as it impacts the bioleaching microorganism’s growth and the microbial communities’ structure, thereby influencing the leaching rate. Many bioleaching microorganisms are acidophiles, and high pH may inhibit their oxidation ability. On the other hand, low pH levels (i.e., lower than 0.8) are also unfavorable to bioleaching because they impede microbial development and oxidative activity. This means microorganisms can be more functional in bioleaching if the medium pH is adjusted appropriately since a deviation from the optimum can have a detrimental impact on their growth and the leaching efficiency. The bioleaching of mineral sulfides mostly occurs at pH < 3 where there is an abundance of proton concentration. This can be attributed to the regeneration of Fe^3+^ at pH < 3 through biological oxidation of Fe^2+^. For example, Guo et al. (2010) reported that a bit of variation of 0.25 in pH (pH 1.25 and 1.5) resulted in a varied amount of As release (Guo et al., 2010). Additionally, the speed and the leaching reaction process are influenced by the initial pH, which strongly affects the bacteria growth, sulfur oxidization, and the rate of toxic metal(loid)s removal (Yu et al., 2014; Fonti et al., 2016; Ghavidel et al., 2018). Typically, a decrease in soil pH increases the bioavailability of metals in soil solution. This is due to the following reasons: at lower pH, the dissolution-precipitation equilibrium among metal ions is broken to release metal ions into the soil solution. Secondly, the exchangeable capacity on the surface of soil particles between metal cations and H^+^ is more significant at lower pH than at higher pH (Sheoran et al., 2016). At pH levels 5, 7, and 9, the influence of pH on heavy metal removal was studied (Yang et al., 2018b). The results indicated that metal removal efficiency decreased initially and subsequently increased with increasing pH value, but the degrees of increase varied. Maximum removal efficiencies of Cd, Zn, Mn, and Cu were at pH 5, while that of Pb and As were at pH 9. The majority of bioleaching fungi are acidophiles (pH 4.0–6.0), although they may also live in severe environments (pH 3.0–9.0; Walker and White, 2017). The metabolism of fungi is affected by a change in pH. For example, more citric acid is produced when the medium pH is <3.0, while secretion of gluconic acid is favored when the medium pH is between 3.0–4.0, and oxalic acid production is also favored when the medium pH is >6.0 (Amiri et al., 2011). Additionally, pH values are chosen not only because of the use of acidophilic microorganisms but also because of decreasing the formation of jarosite (Rouchalova et al., 2020). The microbial community composition is also influenced by pH. Previous research (Wang et al., 2014; Wang Y. et al., 2018) has shown that a reduction in pH during bioleaching increases microbial diversity and abundance. Additionally, relevant research has shown that pH significantly affects the structure of the microbial community and the surface of the minerals at different depths in the column (Yun et al., 2016).

## Redox potential

The redox potential of soil is a measurement of the soil solution’s ability to obtain or release electrons. In the soil, several metal ions are in different redox states, and the oxidation states of metals significantly impact solubility and mobility (Sheoran et al., 2016). Redox potential is critical, and a required component in bioleaching since it determines the metabolic kinds of bacterial communities to a large extent (Husson and Sarthou, 2017). As Fe^2+^ is biooxidized to Fe^3+,^ the redox potential of the culture medium also increases. Higher redox potential is best suited for the leaching of metals, and higher concentrations of Fe^3+^ indicate high oxidation–reduction potentials (Billy et al., 2018; Masaki et al., 2018). The efficient use of Sulfur favors high redox potential in the bioleaching process. However, adding chemical reductants or restricting the oxygen supply can control the redox potential. Controlling redox potential in the initial stage to enhance bioleaching has been reported in many studies. For instance, a high redox potential will improve the bioleaching of pyrite, whereas a low redox potential will favor the bioleaching of chalcopyrite (Petersen and Dixon, 2006; Gericke et al., 2010).

## Temperature

Temperature is an essential factor in bioleaching. Higher reaction temperatures result in a faster reaction rate, and this is one major distinguishing factor between bioleaching and chemical leaching. Temperature, as a physiochemical factor, also affects the microbial community structure. The bioleaching efficiency is enhanced when performed at the optimum temperature of the microorganism (Yu et al., 2019) and temperature modulation is a challenge in chemical leaching. The optimal temperature for bioleaching is determined by the characteristics of the bacteria species used, and organic acids secreted by heterotrophic bacteria during the leaching process are influenced by temperature. Many organic acids are produced and more toxic metal(loid)s leached when the temperature is just appropriate. Microorganism growth and enzyme activity are inhibited when the temperature is too high, and leaching efficiency suffers as a result (Rawlings, 2005; Wei et al., 2018). For example, a higher temperature of 40°C decreased the *Aspergillus flavus* and other *Aspergillus* genus biomass and lowered their leaching efficiency (Qayyum et al., 2019). Lower temperatures in the growth phase also reduce the chances of a successful enzyme-substrate collision. High temperatures, meanwhile, denature the enzymes required for the cell cycle. In bioleaching, the optimal temperature for high metabolic activity from mesophilic bacteria is about 30°C–35°C while thermophilic bacteria could be utilized at 50°C and 80°C (Fonti et al., 2016; Walker and White, 2017). Most *Aspergillus* genus have been used for bioleaching at 30°C (Horeh et al., 2016). For example, *Aspergillus flavus* demonstrated a significant leaching efficiency of Cd, Pb, and Zn from contaminated soil at a temperature of 30°C, as well as producing many organic acids (Qayyum et al., 2019). One of the key elements affecting microbial life is temperature (Gilbert et al., 2012; He et al., 2012). Therefore, it is unsurprising that microorganisms are the most often used models for researching how temperature affects biological processes. The temperature sensitivity of microorganisms also influences bioleaching systems. A column leaching at four different temperatures (30°C, 40°C, 50°C, and 60°C) found only *At. caldus*, *L. ferriphilum,* and *F. acidiphilum* were present in the leachate after the microbial community diversity analysis. Surprisingly, even at 60°C, no thermophile sequences were discovered. Additionally, *At. Caldus* was the most predominant, with *L. ferriphilum* accounting for a smaller percentage (Mutch et al., 2010). In sharp contrast, *L. ferriphilum* was discovered to be dominant at 33°C and 45°C, whereas *At. caldus* was identified at 45°C and 65°C only in trace amounts (Zeng et al., 2010; Chen et al., 2014). In several studies, temperature has been found to influence microorganisms’ generation time and accelerate their growth (Wang et al., 2016; Xiao et al., 2017). In summary, temperature affects the bioleaching process in two ways: promoting the activity of the microorganisms involved in the leaching process and modifying the ratio of bacterial species present throughout the leaching process.

## Oxygen and carbon dioxide supply

Fundamentally, the impact of oxygen in bioleaching cannot be overlooked. Microorganisms playing vital role in metal dissolution in bioleaching need oxygen to speed up the oxidation of sulfide minerals and carbon dioxide as carbon source for the cell growth. Bioleaching requires obligate microbes, so low oxygen concentration negatively affects their metabolic activities and, thus, the oxidation rate (Khainasova, 2019). The microbial community also gets affected by the oxygen and carbon availability in the bioleaching heap, since only microorganism capable of growing under limited conditions are adapted. Oxygen limitation have been reported to cause extended linear growth of sulfur-oxidizing bacteria. Aeration, stirring, or shaking can all be used in the laboratory to alleviate oxygen insufficiency. However, a sufficient oxygen supply may pose a few challenges on a large scale, for example, heap or dump bioleaching. Limited oxygen supply decreases the oxidization reaction rate of SO and Fe^2+^, leading to a delay in the generation of sulfuric acid and metal solubilization. In bioleaching of sediments, for instance, although slow rates of S^0^ and Fe^2+^ oxidation occur even under strong oxygen limitation, oxidation of S^0^ demands elevated levels of oxygen in the presence of sediments, and reduced oxygen availability leads to delay in acidification, sulfate production, and metal solubilization (Rawlings, 2005; Seidel et al., 2006; Fonti et al., 2016). Carbon dioxide availability caused a significant change in the structure of the microbial community and supported the facts that low levels of carbon dioxide are an important factor for microbial population survival and growth in promoting bioleaching (Marín et al., 2017). Petersen et al. (2010) reported on a decline in microbial growth and oxidation as a result of depleted carbon dioxide in bioleaching solutions (Petersen et al., 2010).

## Pulp density

Pulp density (liquid to solid ratio, L/S) is an important variable in the bioleaching process. Pulp density is given as a percentage number that indicates how much solid is present in the solution, and as a result, a higher L/S ratio indicates a lower pulp density (Potysz et al., 2018). Many factors contribute to the negative effects of increasing pulp density, such as the increase in toxicity, which inhibits the metabolic activity of microorganisms (Gu et al., 2018). Additionally, pulp density significantly affects toxic metal(loid)s leaching *via* the influence of pH, and higher pH is proportional to pulp density (Ye et al., 2017). For example, in contact bioleaching at elevated pulp densities, microbial leaching performance is inhibited by releasing toxic compounds and insufficient agitation (Meshram et al., 2016; Marra et al., 2018). Pulp density affects pH, inhibiting microbial growth and reducing the metal extraction rate. The use of bioleaching on a large scale is significantly influenced by pulp density (Liu et al., 2020). A 2% pulp density instead of 1% accounts for a 50% reduction in the quantity of bioleaching solution required, lowering the operating cost (Niu et al., 2014). In a batch reactor, the impact of varied mineral pulp densities of 5, 10, and 30 g/L on bacterial activity was studied. The pH of *A. thiooxidans* increased as the pulp density increased, but the pH of *P. putida* did not alter as the pulp density increased (Bolaños-Benítez et al., 2018). High pulp density is expected to restrict microbial activity, hence a pulp density of 15% had a significant effect on the leaching of copper and Zinc (Guo et al., 2010). As bioleaching approaches industrial scale, optimal pulp density is vital for economic reasons because the process may be hampered by high pulp density, reducing metal recovery. Alternatively, a low pulp density may hinder productivity in large-scale applications. Therefore, a pulp density that balances bioleaching efficiency with economic viability must be identified (Desmarais et al., 2020).

## Growth medium

It has been proven that bioleaching effectiveness is substantially determined by the chemical and mineralogical composition of the soil; hence, restricted nutrients in soils lower bacteria’s growth rate, limiting bioleaching efficiency (Chen and Lin, 2010). Thus, different quantities of nutrients (organic and inorganic) are a prerequisite for microbial growth and metabolite production to enhance bioleaching (Elkina et al., 2021). Synthetic nutrients like (NH_4_)_2_SO_4_, K_2_HPO_4_, and H_3_PO_4_ are frequently used to supply nutrients for microbial growth (Govarthanan et al., 2014). Also, the addition of yeast to the growth medium result in the rapid growth of the bacteria. A rapid initial growth of *A. brierleyi* was observed when yeast was added to the growth medium (Plumb et al., 2008). Depending on the kind of microbe utilized and the projected bioleaching process, several growth medium compositions (nutrient supply) are used. For instance, chemolithoautotrophic bacteria *A. ferrooxidans* utilize ferrous salts as major nutrition to generate sulfuric acid and Fe^3+^ ions, whereas *A. thiooxidans* need elemental sulfur to produce sulfuric acid (Zhang et al., 2018; Quatrini and Johnson, 2019). A high degree of bacterial activity and the concomitant accelerated sulfur oxidation allows for the production of adequate acid to initiate the leaching reaction. Differing concentrations of sulfur supplementation might be used to boost bacteria’s performance (Potysz et al., 2018). To explain this, a study by (Kaksonen et al., 2016) revealed sulfur supplements boost metal extraction efficiency by 20% compared to leaching in settings devoid of elemental sulfur. The usage of sulfur substrate also caused *Acidithiobacillus* and *Sulfuritalea* to dominate the microbial community composition, further enhancing the leaching efficiency (Wu C. et al., 2020). However, a high dose of sulfur inhibits the substrate in the bioleaching process. Furthermore, unused or unoxidized sulfur may cause re-acidification of treated soils during the bioleaching process. As a result, the sulfur dose utilized in bioleaching must be carefully calculated.

## Surfactants and natural extracts

The eco-sustainability, superior contaminant removal efficiency, flexibility, and the green chemistry basis of surfactants have gained them attention for removing contaminants from their different media (Rasheed et al., 2020; Kumar et al., 2022). The surfactant’s unusual molecular structure improves the water solubility of soil pollutants, particularly hydrophobic organic molecules (He et al., 2016). This may be attributed to the decrease in surface tension and reduction of mass transfer of oxygen hence surfactants can speed up and enhance the rate of leaching (Aioub et al., 2019). As the bioleaching system relies on the multi-phase interface interactions between microbes, minerals, solution, and other factors, altering the properties of mineral surfaces and bacterial outer membranes enhances the interface action between microbes and minerals to increase leaching speed (Fang et al., 2014). Different surfactants, including anionic, cationic, zwitterionic, and nonionic, have been tested and/or applied to remove toxic metal(loid)s from contaminated soils. Generally, surfactant adsorption into soils is supposed to be minimal for successful surfactant-enhanced remediation, however, surfactants have a considerable solubilizing activity on the target pollutant and can also be harmful to the microbial community (Mao et al., 2015; Kumar et al., 2021). This contributes to toxic metal(loid)s being removed *via* the surfactant-associated complexation and ion exchange. Besides the positive effect of solubilizing and desorbing soil contaminants, biosurfactants promote microbial decomposition of the contaminants, hence facilitating the removal mechanism. Combining surfactants with other additives, such as organic solvents, chelating agents, and ligand ions can also enhance the removal of contaminants from the soil (Zheng et al., 2012).

## Limitation of the bioleaching technology

The public’s health, the environment, and the economy are all greatly improved by bioleaching technology. This has contributed to it gaining much attention since it is simple to operate, less expensive, lower energy requirement and less toxicity. However, the process constrained with certain limitations. This includes the slow kinetics of the bacterial leaching process. Therefore, it is important develop catalyst to optimize the interactions of the microbes with the minerals while accelerating the kinetics. Also, most studies in respect to bioleaching of contaminated soil is limited to laboratory scale and hence accurate estimation for commercial scale is still in the infancy stage. Furthermore, toxic chemicals are sometimes produced in the process. For example, sulfuric acid and H+ ions generated can seep into the ground and surface water, rendering it acidic and harming the ecosystem. For these reasons, a bioleaching setup must be carefully planned to prevent the process from compromising biosafety.

## Current and future perspectives of omics on bioleaching

Genetics, genomics, metabolomics, and proteomics are novel technology platforms referred to as omics. The use of an omics approach in bioleaching will assist in answering concerns about the complicated role of microorganisms. These techniques will help predict the metabolic models and improve scientific understanding of the physiology of the microorganisms used in bioleaching (Watling, 2016). Additionally, these omics technologies will aid in discovering novel features and characteristics of microorganisms relating to gene, protein, macromolecule, and environmental interactions (Baniasadi et al., 2019). Genomics is currently enhancing our understanding of bioleaching. Through partial and whole-genome sequencing, it has become possible to identify biodiversity within leaching environments and create molecular-based methods for analyzing the temporal dynamics of various bioleaching processes. For a while, it was thought that *Acidithiobacillus ferrooxidans* was the most important bacteria for metal sulfide bioleaching; however, current development in genomics comprising bioidentification molecular techniques such as DGGE, FISH, and quantitative PCR (qPCR), has prompted the search for novel species with potential in extreme mineral leaching environments. The identification of heterotrophic archaea initially classified as *Ferroplasma cupricumulans* and later reclassified as *Acidiplasma cupricumulans*, the moderate thermophilic mix and/or heterotrophs from the genus *Sulfobacillus,* and the chemolithoautotrophic iron-oxidizing *Leptospirilli* are just a few examples of how genomics has been used to show the diversity of microbial populations in bioleaching operations. A high-throughput proteomic study identified 131 proteins in the periplasmic fraction of thiosulfate-grown cells, which has contributed to a better understanding of *Acidithiobacillus ferrooxidans* ATCC 23270^T^ sulfur oxidation metabolism (Chi et al., 2007). Moreover, at present, targeted and untargeted analytical strategies for analysis and detection of metabolites, using capillary electrophoresis and MS for separation and identification, have been used in the first metabolomic study in bacteria using two microorganisms isolated from mining sites in Chile, *Acidithiobacillus ferrooxidans* strain Wenelen, DSM 16786 and *Acidithiobacillus thiooxidans* strain Licanantay, DSM 16786 (Martínez et al., 2013).

One of the essential aspects of bioleaching is biofilm formation. Biofilm cells are immersed in an EPS matrix that aids in the adhesion of biofilm cells to solid surfaces and the subsequent corrosion of those surfaces (Vera et al., 2013). This corroborates with the high spermidine concentrations, especially under sulfur-growth conditions, observed in the supernatants of *Acidithiobacillus ferrooxidans* strain Wenelen and *Acidithiobacillus thiooxidans* strain Licanantay (Martínez et al., 2013). In summary, bioleaching has experienced some success with genomics, proteomics, and metabolomics, although there are still significant gaps in the literature. Due to the sensitivity and imprecision of current methods like mass spectrometry, many proteins are still unable to be identified using proteomics. To address this issue, efforts are being made to replace mass spectrometry with other technologies, such as sub-nanopore array, nanopore 5D fingerprinting, and fluorescent protein fingerprinting. In the study of bioleaching, metabolomics is still in its infancy, and no one analytical technique has adequately accounted for all the metabolic components of environmental materials. Therefore, more sophisticated methods are required to detect every bioleaching microorganism metabolite.

## Conclusion

The accumulation of toxic metal(loid)s in soil, their long-term persistence, and eventual penetration into the food chain can cause significant environmental damage. In the last decade, bioleaching has become a promising technology for removing toxic metals (loids) from contaminated soil and has received much attention due to its economic value and eco-environmental friendliness. Although not much literature has been reported on the use of bioleaching for contaminated soils on a large scale, advancement in the process will enable its use on a larger scale. Additionally, the environmental remediation value of bioleaching will be enhanced if the bioleaching process is used in conjunction with omics, as this will assist in developing novel strains that are applicable for use in large-scale bioleaching of contaminated soil.

## Author contributions

ES: methodology and writing – review and editing. KL, JY, ZG, JS, and YD: writing original – draft. HL, HJ, YL, and HY: review and editing. LJ and XL: conceptualization and supervision. All authors contributed to the article and approved the submitted version.

## Funding

Financial support from the National Key Research and Development Program of China (Grant No. 2018YFC1800400), the National Natural Science Foundation of China (Grant No. 51909282), Natural Science Foundation of Hunan Province of China (Grant No. 2022JJ40583) and Hunan Provincial Key Research and Development Plan (Grant No. 2022WK2017) is highly appreciated.

## Conflict of interest

The authors declare that the research was conducted in the absence of any commercial or financial relationships that could be construed as a potential conflict of interest.

## Publisher’s note

All claims expressed in this article are solely those of the authors and do not necessarily represent those of their affiliated organizations, or those of the publisher, the editors and the reviewers. Any product that may be evaluated in this article, or claim that may be made by its manufacturer, is not guaranteed or endorsed by the publisher.
